# The role of auxin transporters in monocots development

**DOI:** 10.3389/fpls.2014.00393

**Published:** 2014-08-15

**Authors:** Sara Balzan, Gurmukh S. Johal, Nicola Carraro

**Affiliations:** ^1^Department of Agronomy, Animals, Food, Natural Resources and Environment, Agripolis, University of PadovaPadova, Italy; ^2^Department of Botany and Plant Pathology, Purdue UniversityWest Lafayette, IN, USA; ^3^Department of Agronomy, Purdue UniversityWest Lafayette, IN, USA

**Keywords:** IAA, PIN, ABCB, AUX/LAX, PILS, PAT

## Abstract

Auxin is a key regulator of plant growth and development, orchestrating cell division, elongation and differentiation, embryonic development, root and stem tropisms, apical dominance, and transition to flowering. Auxin levels are higher in undifferentiated cell populations and decrease following organ initiation and tissue differentiation. This differential auxin distribution is achieved by polar auxin transport (PAT) mediated by auxin transport proteins. There are four major families of auxin transporters in plants: PIN-FORMED (PIN), ATP-binding cassette family B (ABCB), AUXIN1/LIKE-AUX1s, and PIN-LIKES. These families include proteins located at the plasma membrane or at the endoplasmic reticulum (ER), which participate in auxin influx, eﬄux or both, from the apoplast into the cell or from the cytosol into the ER compartment. Auxin transporters have been largely studied in the dicotyledon model species *Arabidopsis*, but there is increasing evidence of their role in auxin regulated development in monocotyledon species. In monocots, families of auxin transporters are enlarged and often include duplicated genes and proteins with high sequence similarity. Some of these proteins underwent sub- and neo-functionalization with substantial modification to their structure and expression in organs such as adventitious roots, panicles, tassels, and ears. Most of the present information on monocot auxin transporters function derives from studies conducted in rice, maize, sorghum, and *Brachypodium*, using pharmacological applications (PAT inhibitors) or down-/up-regulation (over-expression and RNA interference) of candidate genes. Gene expression studies and comparison of predicted protein structures have also increased our knowledge of the role of PAT in monocots. However, knockout mutants and functional characterization of single genes are still scarce and the future availability of such resources will prove crucial to elucidate the role of auxin transporters in monocots development.

## INTRODUCTION

Plants exhibit an astonishing variety of shapes and develop multicellular bodies able to live for hundreds of years and reach considerable size. They rely on continuous growth and are able to regenerate organs from undifferentiated meristematic cells populations. Plant growth and organ differentiation, as well as response to environmental stimuli, are regulated, among other factors, by endogenous compounds called phytohormones. They control the plant developmental program by regulating cell division and expansion, tissue differentiation, and senescence. Phytohormones can act within the cell of origin or move to other sites in the plant, where they are perceived as a signal by hormone receptors (Davies, 2004).

The plant hormone auxin was first isolated as Indol-3-acetic acid (IAA) by Went (1926), as he studied the tropic response of *Avena sativa* coleoptiles. Subsequently, during the first half of the twentieth century, other four phytohormones were identified, including abscisic acid, cytokinins, gibberellins, and ethylene (Kende and Zeevaart, 1997). More recently, several additional compounds have been recognized as hormones including brassinosteroids (BR), jasmonate (JA), salicylic acid (SA), nitric oxide (NO), and strigolactones (SLs) (Tarkowská et al., 2014). Auxin is a regulator of many aspects of plant development, including cell division, elongation, differentiation, embryonic development, root and stem tropisms, apical dominance, and flower formation (Young et al., 1990; Woodward and Bartel, 2005; Tanaka et al., 2006; Möller and Weijers, 2009; Leyser, 2010; Müller and Leyser, 2011; Christie and Murphy, 2013; Gallavotti, 2013; Geisler et al., 2014). Besides IAA, which is the most abundant natural form of auxin, several auxin-like molecules have been identified. While 4-chloroindole-3-acetic acid (4-Cl-IAA), indole-3-butyric acid (IBA), and phenylacetic acid (PAA) are all found in plants, 2,4-dichlorophenoxyacetic acid (2,4-D) and naphthalene-1-acetic acid (NAA) are synthetic compounds that have biological activity similar to IAA (Bertoni, 2011; Simon and Petrášek, 2011).

**FIGURE 1:**
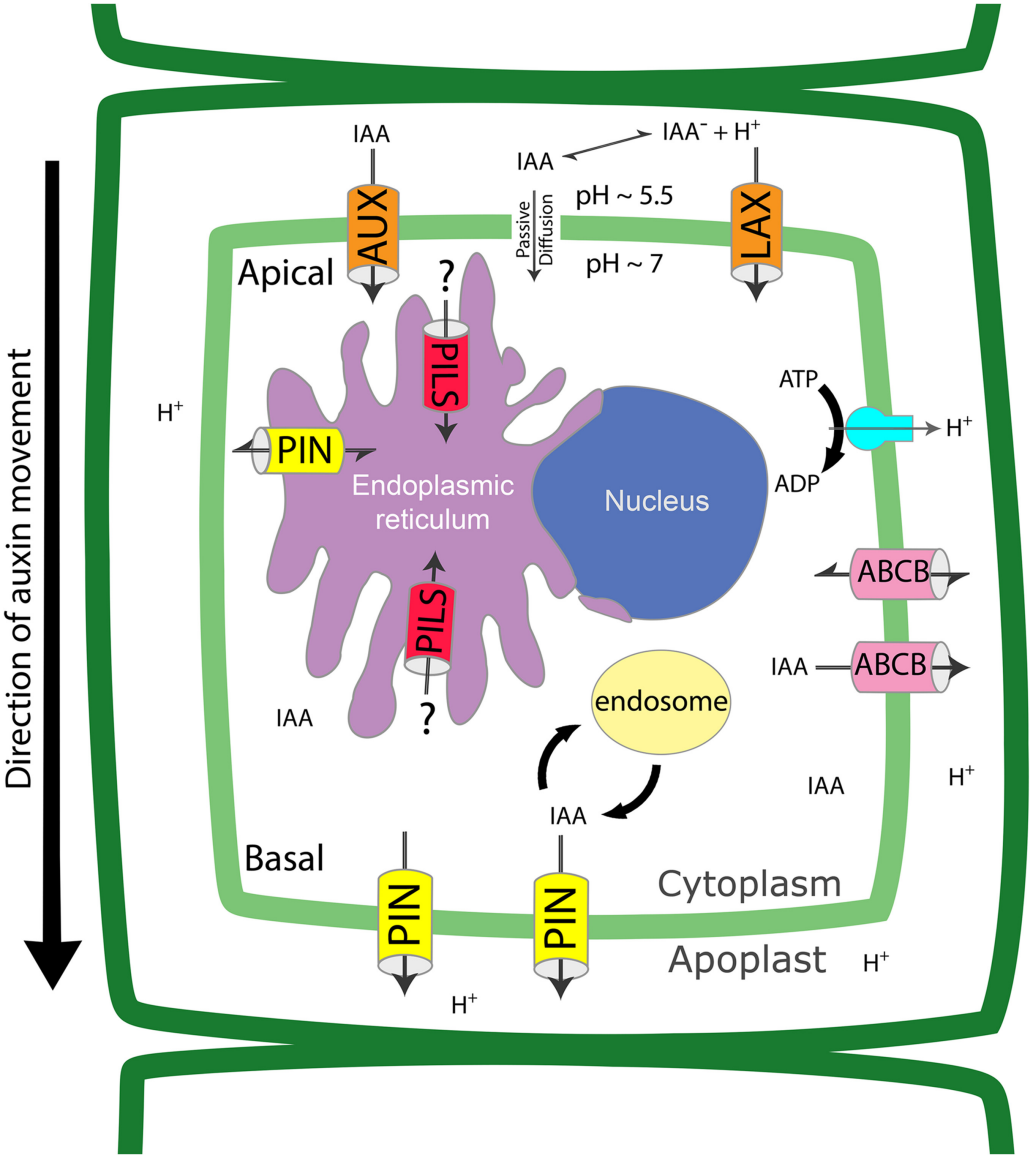
**Auxin transport proteins regulate intracellular and cell to cell auxin fluxes.** Auxin (IAA) crosses the plasma membrane through passive diffusion, as protonated form, or through PM transporters, as deprotonated form. PINs are eﬄux carriers located at the PM and ER and can be re-inserted in the lipid bilayer by recycling via the endocytic pathway. AUX/LAXs and PILs are influx carriers located at PM and ER, respectively. ABCBs are located at the PM and use energy from ATP to traslocate IAA. The coordinated localization of the different transporters determines the overall directionality of the auxin flux and contributes to the regulation of intracellular auxin levels.

Local biosynthesis, degradation and conjugation contribute to the modulation of IAA homeostasis at the cellular level. Availability of free IAA inside the cell is also controlled by auxin transport, which occurs in two distinct pathways: a passive diffusion through the plasma membrane (PM) and an active cell-to-cell transport, depending on the protonation state of IAA. IAA is a weak acid with a dissociation constant of p*K* = 4.8. In a neutral or basic environment IAA^-^ will be the most abundant form (99.4% ionized at pH = 7.0), whereas in the acidic extracellular space IAAH is predominant (about 20% protonated at pH = 5.5) (Delbarre et al., 1996; Estelle, 1998; Kramer and Bennett, 2006). IAAH can enter into the cell through the PM by passive diffusion or active transport by PM importers. Once inside the cytoplasm, which has a neutral pH, IAA^-^ becomes the predominant form and it cannot freely move out of the cell unless actively transported by eﬄux carrier proteins (**Figure 1**). The differential localization of transporters at specific sites on the PM creates a directional auxin flow that eventually establishes a polar auxin transport (PAT) stream through adjacent cells. Four classes of auxin transporters have been identified: the PIN-FORMED (PIN) exporters, the ATP-binding cassette (ABC)-B/multi-drug resistance/P-glycoprotein (ABCB/MDR/PGP) subfamily of ABC transporters, the AUXIN1/LIKE-AUX1 (AUX/LAX) importers, and the newly described PIN-LIKES (PILS) proteins.

Despite the fact that auxin was first isolated and studied in the monocot *A. sativa,* characterization of auxin transport proteins derives mostly from forward genetic studies of mutants with defects in development, organ morphogenesis, and gravitropism in the dicot *Arabidopsis thaliana*. In recent years, the number of studies on the biological role of PAT in monocots has increased. This has been facilitated by the lower cost of deep sequencing of whole plant genomes and transcriptomes and by the availability of tools such as transgenic lines carrying proteins with fluorescent tags, which are used in subcellular localization studies and PAT fluxes modeling (Mohanty et al., 2009; Egan et al., 2012; Yu et al., 2012). In this work, we present a comprehensive description of monocots auxin transporters and provide, where possible, functional comparison between monocot and *Arabidopsis* proteins.

## MATERIALS AND METHODS

PIN and PILS protein sequences of *Arabidopsis*, rice, maize sorghum, and *Brachypodium* (gene accession numbers are listed in Table S1) were aligned using the CustalW 2.0 software (Larkin et al., 2007). The alignment file was used to generate an unrooted tree with MEGA 6.0 (Tamura et al., 2013), applying the Neighbor-joining method, the Poisson model and 500 bootstrap replications. Bootstrap analysis values >60 are indicated at each node.

## PINs

PINs are the most studied family of auxin transporters in plants. *PIN* genes are present in eight copies in *Arabidopsis* and encode integral membrane proteins with two conserved domains formed by transmembrane helices, typically five at both the N and C termini, and a less conserved central hydrophilic loop of variable length (Křeček et al., 2009; Ganguly et al., 2012). Their subcellular localization has been correlated with the length of the hydrophilic domain. In *Arabidopsis*, PIN1, -2, -3, -4, and -7 have a longer loop (ranging in size from 298 to 377 amino acid residues), PIN5 and -8 have a shorter loop (27–46 residues) and PIN6 contains an intermediate form (Křeček et al., 2009; Ganguly et al., 2010; Viaene et al., 2013). “Long” PINs are generally inserted into the PM while “short” PINs are located in the endoplasmic reticulum (ER) and they are thought to contribute to intracellular auxin homeostasis (Mravec et al., 2009; Ding et al., 2012; Cazzonelli et al., 2013). More recently, it has been demonstrated that PIN5 is also PM localized, depending on the cell type and developmental stage, and that PIN5, -6, and -8 function in polar cell-to-cell transport of auxin by regulating coordinated influx and eﬄux of IAA into and out of the ER (Bender et al., 2013; Sawchuk et al., 2013; Ganguly et al., 2014).

Several auxin transporters show polar localization in the cell, but it is only in the case of PIN proteins that polar targeting occurs more frequently (**Figure 1**). Shifting PIN polarity results in the alteration of PAT which leads to developmental defects in *Arabidopsis* (Löfke et al., 2013). The polar localization of PIN proteins is established by cycling between the PM and endosomal compartments such as the trans-Golgi network/early endosomes (TGN/EE). PIN recycling can take place *via* endocytosis of clathrin-coated vesicles and depends on phosphorylation and ubiquitination (Robert et al., 2010; Kleine-Vehn et al., 2011; Löfke et al., 2013). Unphosphorylated PINs, or those dephosphorylated by the PP2A/PP6 phosphatase, are recycled to the PM by the brefeldin A (BFA)-sensitive ADP-ribosylation factor-guanine nucleotide exchange factor (ARF-GEF) GNOM. Phosphorylation of PIN proteins by the protein kinase PINOID (PID) results in their GNOM-independent recycling to the PM on the opposite side of the cell (Friml et al., 2004; Dhonukshe et al., 2010). Monoubiquitination and subsequent polyubiquitination of PIN proteins induce their endocytosis, followed by trafficking from the TGN/EE to late endosomes, from where the SNX1/BLOC-1 complex mediates transfer to multivesicular bodies (MVBs) for vacuolar degradation (Habets and Offringa, 2014). Recently, another *Arabidopsis* kinase, D6 PROTEIN KINASE (D6PK), has been demonstrated to regulate PIN phosphorylation and, together with PID, D6PK promotes PINs-mediated auxin transport at the PM by maintaining their phosphorylation status. D6PK PM localization is essential to establish and maintain PIN phosphorylation, and *d6pk* mutants have defects in both negative gravitropism and phototropism due to impaired auxin transport (Zourelidou et al., 2009; Willige et al., 2013; Barbosa et al., 2014).

Phylogenetic studies on the origin and evolution of PIN proteins have demonstrated that their general structure is highly conserved across the plant kingdom and suggest that the last common ancestor of land plants had at least one “long” (canonical) PIN protein (Carraro et al., 2012; Bennett et al., 2014). Strong selective pressure maintained PINs function as auxin carriers while they underwent sub- and neo-functionalization with substantial modification to protein structure, possibly due to selective loss of phosphorylation sites in their central loop (Dhonukshe et al., 2010; Fozard et al., 2013; Bennett et al., 2014). This generated several clades of non-canonical proteins with shorter and divergent structure, leading to altered localization and biological function. Monocot PIN families are often enlarged due to whole genome duplications and the retention of multiple copies of similar proteins. Both *Oryza sativa* and *Zea mays* contain four *PIN1* copies and present at least one monocot-specific gene, *PIN9,* which is divergent in sequence and expression pattern from the closest dicot *PINs* (Xu et al., 2005; Forestan et al., 2012; Bennett et al., 2014; Clouse and Carraro, 2014). The PIN9 protein profile prediction shows an intermediate hydropathic profile in between the “long” and the “short” PINs.

*AtPIN1* is expressed early during embryonic development and later both in the primary root and in the inflorescence stem. Disruption of *AtPIN1* expression leads to the formation of naked, pin-shaped inflorescences and abnormalities in the number, size, shape, and position of lateral organs (Okada et al., 1991; Gälweiler et al., 1998). As suggested by the *pin1* phenotype, PIN1 plays an important role in establishing the plant developmental plan and is involved in floral bud formation, phyllotaxis (the arrangement of leaves and flowers around the stem), vascular development, vein formation, embryogenesis, lateral organ formation, anther development, and root negative phototropism (Gälweiler et al., 1998; Benková et al., 2003; Reinhardt et al., 2003; Weijers et al., 2005; Feng et al., 2006; Scarpella et al., 2006; Lampugnani et al., 2013; Zhang et al., 2014). *Arabidopsis pin2* was the first *pin* mutant identified in a screen for agravitropic seedlings by Bell and Maher (1990). Initially, it was called *agr1*, and the gene responsible for the phenotype was cloned independently by four research groups and named *AGR1/EIR1/PIN2/WAV6* (Chen et al., 1998; Luschnig et al., 1998; Müller et al., 1998; Utsuno et al., 1998). AtPIN2 functions in auxin-regulated root gravitropic response and its expression levels and polar cellular localization are altered by salt stress (Abas et al., 2006; Sun et al., 2008). *AtPIN3* is expressed during embryo development and the *Atpin3* mutant shows reduced growth, and decreased apical hook formation (Friml et al., 2002b; Zádníková et al., 2010). It has been shown that *AtPIN3* plays an important role in root gravitropism, as in vertically grown seedlings AtPIN3 is positioned symmetrically at the PM in the columella cells, but rapidly re-localizes laterally to the lower PM of the statocytes following gravistimulation. AtPIN3 relocalization determines the direction of the auxin flux, which leads to asymmetric auxin accumulation and subsequent differential cell growth (Friml et al., 2002b). AtPIN3 is also involved in root negative phototropic response, as blue-light induced AtPIN3 polarization is needed for asymmetric auxin distribution (Zhang et al., 2013).

AtPIN4, as well as AtPIN7, are involved in auxin controlled embryo, primary root, and apical hook development (Friml et al., 2002a, 2003; Vieten et al., 2005; Kleine-vehn et al., 2010; Zádníková et al., 2010). AtPIN7 also undergoes relocalization similar to AtPIN3 in response to gravistimulation in a subgroup of the columella cells (Rosquete et al., 2013). AtPIN5 is involved in auxin homeostasis, and it has been demonstrated that it can export auxin from yeast cell (Mravec et al., 2009). AtPIN6 is involved in stamen development, and microarray and reporter assays have demonstrated that it is necessary for nectaries development (Bender et al., 2013). Expression analysis of *pPIN6::PIN6-GFP* lines during leaf development has demonstrated that AtPIN6 localizes to the ER and expression is initiated in broad sub-epidermal domains that later on narrow to sites of vein formation (Bender et al., 2013; Sawchuk et al., 2013). Moreover, AtPIN6 is implicated in processes such as shoot apical dominance, lateral root primordia development, adventitious root formation, root hair outgrowth, and root waving where it regulates auxin homeostasis (Cazzonelli et al., 2013). *AtPIN8* is expressed in the male gametophyte, and has a crucial role in pollen development and functionality (Ding et al., 2012). *AtPIN8*, together with *AtPIN5* and *AtPIN6*, also take part into leaf vein network patterning by regulating intracellular auxin transport between the cytoplasm and the ER lumen. Their action is exerted coordinately with *AtPIN1*, in order to modulate intracellular auxin levels in extending veins (Sawchuk et al., 2013).

### PINs IN *Oryza sativa*

Rice has 12 *PINs* (Table S1) and *OsPIN1* was first described by Xu et al. (2005). Transmembrane motif analysis of the deduced amino acid sequence shows that OsPIN1 is a “long” (canonical) PIN protein, which harbors a long hydrophilic loop and two transmembrane regions composed of five helices each. Protein structure, phylogenetic, and functional analysis identify OsPIN1 as the closest ortholog of AtPIN1 (Xu et al., 2005; Carraro et al., 2012; Wang et al., 2014a). The three *OsPIN1s*, *-a*, *-b*, and *-c* are expressed in roots, stem base, stem and, at a lower level, in leaves and young panicles. In these last two organs, *OsPIN1c* expression is lower than in the other tissues (Xu et al., 2005; Wang et al., 2009). Over-expression of *OsPIN1* in *35S::OsPIN1* transgenic plants increases primary root length and lateral root number. Suppression of *OsPIN1* expression obtained by RNA interference (RNAi) reduces the number of adventitious roots and increases the number of tillers and the tiller angle. Thus, OsPIN1 is involved in auxin transport in primary and adventitious roots, which are more abundant in rice compared to *Arabidopsis.* The role of OsPIN1 in PAT was confirmed by treating wild type plants collars with 1-*N*-Naphthylphthalamic acid (NPA), which blocks initiation and growth of adventitious and lateral roots, while application of the auxin NAA rescues the RNAi-induced phenotype (Xu et al., 2005). *OsPIN2* is the most recently characterized *PIN* gene of rice and shows a different expression pattern compared to *OsPIN1* (Chen et al., 2012). Wang et al. reported that *OsPIN2* is highly expressed in roots and at the base of the stem, less in young panicles and exhibits no expression in adult stem and leaves (Wang et al., 2009). Over-expression of *OsPIN2* results in a larger tiller angle, reduced plant height and an increase in tillers number compared to wild type. *OsPIN2* over-expression increases auxin transport from the shoot to the root–shoot junction and transgenic plants are less sensitive to root growth inhibition by NPA (Chen et al., 2012). Overall, the results indicate that *OsPIN2* acts in a specific auxin-dependent pathway which includes *OsPIN1b* and *OsTAC1* (*TILLER ANGLE CONTROL 1*), and controls rice shoot rather than root architecture (Chen et al., 2012). Three *OsPIN5* homologs are present on chromosomes 1, 8, and 9 of rice. The expression patterns of *OsPIN5a* and *OsPIN5c* are very similar: while only weakly expressed in roots, they show very high expression levels in leaves, shoot apex, and panicle. Small amounts of *OsPIN5b* transcript are detected in the shoot apex, roots of 6-week-old plants and 4-week-old callus tissue (Wang et al., 2009; Miyashita et al., 2010). One *AtPIN8* homolog has been identified in rice but it has not been characterized yet (Miyashita et al., 2010). *OsPIN9* is highly expressed in adventitious root primordia and pericycle cells at the base of the stem (Wang et al., 2009). *OsPIN9* expression levels in roots are decreased by IAA and increased by cytokinin [6-benzylaminopurine (6-BA)] application (Wang et al., 2009; Shen et al., 2010). Expression analysis shows that *OsPIN10a* is present in the stem, leaves, and young panicle, but not in the roots. *OsPIN10b* is mainly expressed in leaves but also at the stem base and in lateral root primordia and both genes are up-regulated by IAA, 6-BA, and JA treatments (Wang et al., 2009).

### PINs IN *Zea mays*

The *PIN* family in maize includes 12 members characterized by often overlapping but sometimes organ-specific expression domains (Forestan et al., 2010). ZmPINs consist of both “long” (ZmPIN1a, -b, -c, -d, ZmPIN2, ZmPIN10a, -b) and “short” forms (ZmPIN5a, -b, -c, ZmPIN8), with ZmPIN9 having a protein structure in between the two classes (Forestan et al., 2012; Table S1). The *ZmPIN1* homologs were among the first identified in maize and are expressed at the PM, supposedly functioning in PAT at different stages of plant development. The *ZmPIN1a*, *-b*, and *-c* loci are located in duplicated regions on chromosomes 9, 5, and 4, respectively, and, along with *ZmPIN1d*, are characterized by a six exons/five introns gene structure. This gene organization is similar to that of *AtPIN1* and *OsPIN1*. The proteins encoded by *ZmPIN1a*, *-b*, *-c*, and *-d* present higher sequence similarity to AtPIN1 than other PINs from *Arabidopsis* and they should be considered as different orthologs of AtPIN1. In monocot species such as maize, the presence of paralogs encoding protein isoforms derived from duplication and neo-functionalization cannot be ruled out, but so far there is no conclusive evidence in the case of *ZmPINs*. *ZmPIN1a*, *-b*, *-c* are ubiquitously expressed but differentially modulated in maize vegetative and reproductive tissues and during kernel development. ZmPIN1 plays an important role during embryogenesis, where detectable hormone activity inside the developing maize embryo appears much later than in *Arabidopsis* (Forestan et al., 2010; Chen et al., 2014). *In situ* hybridization showed that *ZmPIN1a* localizes in the root apical meristem (RAM) and the calyptrogen, which is a specialized layer of meristematic cells that continuously generate replacements for the root cap cells that die during primary root growth. Immunolocalization experiments locate ZmPIN1 in the central cylinder, vasculature, and cortex of the primary root (Carraro et al., 2006; Forestan et al., 2012). NAA application to a *ZmPIN1a-YFP* reporter line causes a more diffuse localization of ZmPIN1a and leads to changes in root anatomy, reducing the size of both root cap and meristem and developing of a pluristratified epidermis (Forestan et al., 2012). ZmPIN1a also interacts with KNOTTED1 (KN1) in shaping leaves and leaf veins patterns and regulates PAT during ear, tassel, and spikelet differentiation (Carraro et al., 2006; Gallavotti et al., 2008; McSteen, 2010; Bolduc et al., 2012). *ZmPIN1a* was shown to rescue the *Atpin1* phenotype and the application of NPA to plants at different stages of development leads to PAT disruption related defects (Gallavotti et al., 2008; Gallavotti, 2013). *ZmPIN1b* is mainly expressed in the epidermis, root cap, and vasculature. *ZmPIN1c* localizes in the epidermis and vasculature of the root central cylinder, while *ZmPIN1d* is specifically expressed in the tassel, ear, and in the fifth node of adult plants. *ZmPIN1d* is also expressed in the L1 layer of the shoot apical meristem (SAM) and inflorescence meristem during the transition to flowering (Forestan et al., 2012). *ZmPIN2* is expressed in the root tip, male and female inflorescences and is involved in kernel development (Forestan et al., 2012). Interestingly, *ZmPIN2* is up-regulated in the roots of the *brachytic2* mutant, which is characterized by reduced shoot-ward auxin transport at the root apex and reduced root gravitropic growth (McLamore et al., 2010). *ZmPIN5a* is highly expressed in roots and *ZmPIN5b* is expressed in the 5th node of the stalk. *ZmPIN8* is up-regulated during the early phase of kernel development and in the 7th and 8th internodes. *ZmPIN9* is expressed in the root epidermis and pericycle and NAA treatment increases its transcript levels in the root segment just before the root apex. There are two *PIN10* homologs in maize, *ZmPIN10a* and *ZmPIN10b*. Both genes are expressed in the male inflorescence, with *ZmPIN10a* also up-regulated during the early phases of kernel development (Forestan et al., 2012).

### PINs IN *Sorghum bicolor*

In *Sorghum bicolor*, *PINs* have been identified and their expression pattern described, but results from functional analysis are still missing (Shen et al., 2010; Wang et al., 2011). The nomenclature for sorghum *PIN* genes does not match the one followed for *Arabidopsis*, rice, and maize therefore, we include the gene identifier together with the common name (Table S1). In sorghum, “long” PIN proteins have a conserved canonical architecture, with two hydrophobic domains divided by a hydrophilic loop (Zazímalová et al., 2007; Shen et al., 2010). Their chromosomal distribution, expression profile and up- or down-regulation following treatment with auxin transport inhibitors [NPA, 1-naphthoxyacetic acid (1-NOA) and 2,3,5-triiodobenzoic acid (TIBA)] have been described (Shen et al., 2010). The *SbPIN1* (Sb02g029210) transcript, similar to *ZmPIN5c*, is predicted to localize to the tonoplast and to be constitutively expressed in all tissues (Shen et al., 2010). SbPIN2 (Sb03g029320), one of the sorghum proteins predicted to be located at the PM, shows high sequence similarity to ZmPIN10a. *SbPIN3* (Sb03g032850), similar to *At/Os/ZmPIN8*, is highly expressed in flowers (Shen et al., 2010). *SbPIN4* (Sb03g037350) is the sorghum gene that shares most similarity with *ZmPIN9* and it is also highly expressed in roots, although not exclusively (Shen et al., 2010). However, its expression is down-regulated by IAA application and increased by BR, while *ZmPIN9* is up-regulated by auxin treatments (Shen et al., 2010; Forestan et al., 2012). *SbPIN5* (Sb03g043960), similar to *Zm/OsPIN5a*, is expressed at low levels in untreated plants while IAA treatment suppresses expression in leaves and roots (Shen et al., 2010). The gene named *SbPIN6* (Sb04g028170) encodes a “long” form similar to PIN1 proteins (Wang et al., 2011). The gene named *SbPIN8* (Sb07g026370) is the most similar to *ZmPIN5b* having a predicted protein structure of a “short” PIN (Shen et al., 2010). Protein sequence alignment and expression pattern of *SbPIN*9 (Sb10g004430) suggest homology to *ZmPIN10b*. The *SbPIN11* (Sb10g026300) sequence is orthologous to *Zm/OsPIN2* and is more expressed in roots and seedling shoots.

### PINs IN *Brachypodium distachyon*

In the genome of the grass *Brachypodium distachyon* there are both “long” and “short”/“intermediate” PIN forms (Bennett et al., 2014; O’Connor et al., 2014; Wang et al., 2014a). Two *PIN1* paralogs have been identified: *BdPIN1a* (Genebank ID XM_003563990.1) and *BdPIN1b* (Genebank ID XM_003570618.1). *BdPIN1a* and *BdPIN1b* are highly expressed in internal tissues, with *BdPIN1b* spanning a broader domain. Transgenic *Brachypodium* lines carrying *pPIN1a:PIN1a-YFP* and *pPIN1b:PIN1b-YFP* constructs show expression in developing spikelets, suggesting a role in vascular patterning (O’Connor et al., 2014). The newly identified “*Sister-of-PIN1*” (*SoPIN1*)/*PIN11* clade contains *Brachypodium* genes that are divergent in sequence from *BdPIN1s* and have no representatives in *Brassicaceae*. *SoPIN1* is highly expressed in the stem epidermis and is consistently polarized toward regions of high expression of the DR5 auxin-signaling reporter, which suggests a role in the localization of new primordia (O’Connor et al., 2014).

## ABCBs

The ABC superfamily of membrane proteins includes more than a hundred different members in plants (Kang et al., 2011). The subfamily B (ABCB) includes homologs of the mammalian MDRs/PGPs, several of which are involved in auxin transport (Geisler and Murphy, 2006; Cho and Cho, 2013). ABCB transporters are integral membrane proteins that actively transport chemically diverse substrates across the lipid bilayers of cellular membranes (**Figure 1**). The core unit of a functional ABC transporter consists of four domains: two nucleotide-binding domains (NBDs) and two transmembrane domains (TMDs). The two NBDs unite to bind and hydrolyze ATP, providing the driving force for transport, while the TMDs are involved in substrate recognition and translocation across the membrane (Jasinski et al., 2003; Higgins and Linton, 2004; Bailly et al., 2011). *Arabidopsis* has 22 ABCBs and the first ABCBs characterized as functioning in IAA traslocation were identified in seedlings (Sidler et al., 1998; Noh et al., 2001). ABCB1, ABCB4, ABCB14, ABCB15, ABCB19, and ABCB21 are associated with auxin transport, although not exclusively (Geisler and Murphy, 2006; Titapiwatanakun and Murphy, 2009; Kaneda et al., 2011; Kamimoto et al., 2012; Cho and Cho, 2013). To date, the best-characterized ABCBs are AtABCB1, AtABCB4, and AtABCB19. They all function in auxin driven root development and require the activity of the immunophilin TWISTED DWARF1 (TWD1)/FKBP42 to be correctly inserted at the PM (Wu et al., 2010). *AtABCB1/PGP1* was the first plant *MDR*-like gene cloned from *Arabidopsis* and it is localized at the PM in the root and the shoot apex of seedlings (Dudlers and Hertig, 1992; Noh et al., 2001). The *Atpgp1* original mutant exhibits only a subtle phenotype compared to wild type plants, but a new allele designated as *atpgp1-2*, shows a shorter hypocotyl and dwarf phenotype under long-day conditions (Geisler et al., 2005; Ye et al., 2013). Disruption of *AtABCB19/AtMDR1* expression results in partial dwarfism and reduced PAT in hypocotyls and inflorescences (Noh et al., 2001). AtABCB19 functions together with AtABCB1 in long distance transport of auxin along the plant main axis in coordination with AtPIN1, and regulates root and cotyledon development and tropic bending response (Lin and Wang, 2005; Bandyopadhyay et al., 2007; Rojas-Pierce et al., 2007; Nagashima et al., 2008; Lewis et al., 2009; Christie et al., 2011). AtABCB4 is a root-specific transporter involved in auxin transport during root gravitropic bending, root elongation, and lateral root formation (Santelia et al., 2005; Terasaka et al., 2005; Kubeš et al., 2011; Cho et al., 2012). This transporter is substrate-activated and functions as an auxin importer at low substrate concentration, switching to auxin export as the availability of auxin increases (Yang and Murphy, 2009; Kubeš et al., 2011). *AtABCB21* encodes a protein that is the closest homolog to AtABCB4 and is expressed in the aerial parts of the seedling and in the root pericycle cells. Just like AtABCB4, AtABCB21 functions as a facultative importer/exporter that controls cellular auxin levels (Kamimoto et al., 2012). AtABCB14 was first described as a malate importer that functions in the control of stomata aperture according to CO_2_ levels (Lee et al., 2008). More recently, AtABCB14 and 15 have been shown to be active in the vascular tissue of the primary stem, which shows anatomical alterations in *abcb14* and *abcb15* mutants. Since IAA transport along the inflorescence is reduced in both mutants, it was proposed that AtABCB14 as well as AtABCB15 participate in auxin transport (Kaneda et al., 2011).

### ABCBs IN *Oryza sativa*

Homologs of *ABCBs* have been described in monocots. In rice, Garcia et al. identified 24 putative ABCB sequences, with *OsABCB22* and *OsABCB14/16* being homologs of *AtABCB19* and *AtABCB1*, respectively (Garcia et al., 2004; Knöller et al., 2010). *OsABCB14* is expressed in all plant organs, including roots, stem, leaves, nodes, root-stem transition region, filling seeds, panicle, and flowers (Xu et al., 2014). Spatial expression analysis shows that *OsABCB14* expression is higher in root tips than in the basal root zone. Knockout mutants of *OsABCB14* have decreased PAT rates, conferring insensitivity to 2,4-D and IAA. A role for OsABCB14 in auxin uptake and iron (Fe) homeostasis has been demonstrated. Acropetal auxin transport in rice *abcb14* plants root system is significantly lower than in wild type. The iron concentrations in shoots, roots, and seeds are significantly enhanced, and the expression level of iron deficiency-responsive genes was significantly upregulated in rice *abcb14* mutants (Xu et al., 2014). Recent evidence also suggests that *N-*glycosylation of ABCB proteins in rice might be important for root development. In an EMS-generated mutant line for *OsMOGS*, which encodes a mannosyl-oligosaccharide glucosidase, root PAT is altered due to under-glycosylation of OsABCB2 and OsABCB14 (Wang et al., 2014b).

### ABCBs IN *Zea mays* AND *Sorghum bicolor*

**FIGURE 2:**
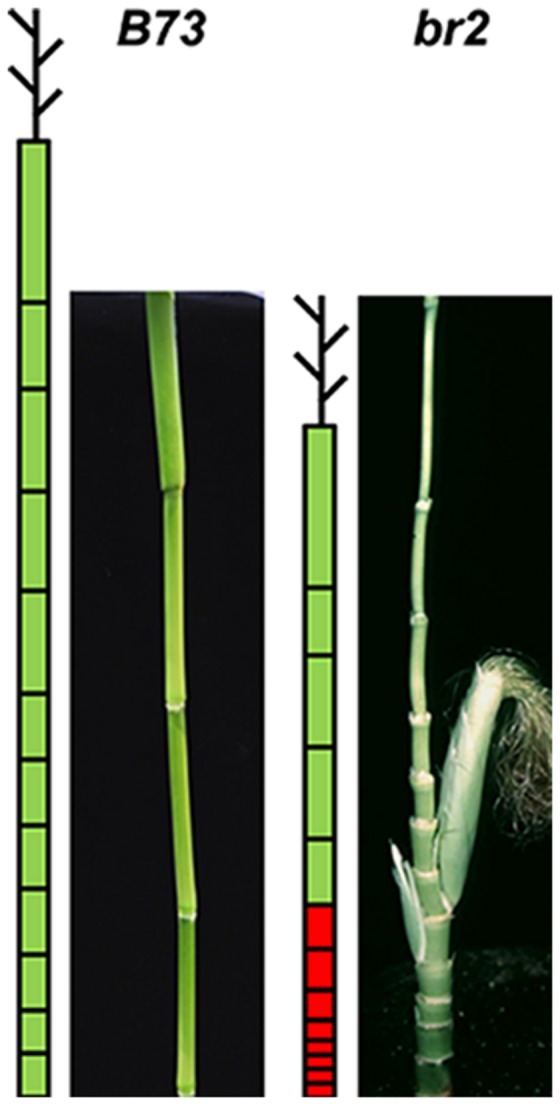
**The *br2* maize mutant shows dramatically impacted stalk architecture.** The *br2* adult plant shows altered stalk height due to reduction in internode length, which is caused by the disruption of IAA transport mediated by ZmABCB1. The same phenotype is present in the sorghum *dw3* mutant, which carries a tandem duplication in the *SbABCB1/Dw3* gene.

In maize and sorghum, loss-of-function mutations in the *AtABCB1* orthologous genes *ZmABCB1* and *SbABCB1* result in short stature plants designated as *brachytic2 (br2)* and *dwarf3* (*dw3*), respectively (Multani et al., 2003). *br2* and *dw3* are characterized by reduced basipetal auxin transport and greatly reduced stalk height (Multani et al., 2003). BR2 is expressed in nodal meristems, and analyses of auxin transport and content indicate that BR2 function in monocot-specific meristems is the same as that of AtABCB1, which is an auxin transporter. Thus ABCB1/BR2 auxin transport ability is conserved between dicots and monocots, but should be considered in the context of distinct architectures of monocot versus dicot plants, which have unsegmented (*Arabidopsis*) and segmented stems (maize, rice, sorghum, *Brachypodium*) (**Figure 2**; Multani et al., 2003; Knöller et al., 2010). The dwarfing phenotype of *dw3* is very similar to that of *br2* and it is the result of a 882-bp tandem duplication in exon 5 that disrupts protein function and the plant’s ability to establish an auxin flux in the intermediate internodes (Multani et al., 2003). These mutants are of particular interest because of the agronomic importance in terms of their ability to resist to lodging and to dramatically enhance the harvest index of the plant. Thus dwarfing traits are important due to the potential distribution of nutrients and energy to grain production rather than vegetative growth. Given that *br2*, which has a defect in *ZmABCB1*, causes the stunting of lower internodes mostly, it raises the possibility that other *brachytic* mutants may arise from defects in other ABCB transporters. In maize, there are three putative *AtABCB19* homologs: *ZmABCB10-1* (GRMZM2G125424) and *ZmABCB2-1* (GRMZM2G072850), present closest sequence similarity to *OsABCB16*, while *ZmABCB10-2* (GRMZM2G085236), is more similar to the true auxin transporter *OsABCB14* (Knöller et al., 2010). *ZmABCB10-1* (GRMZM2G125424) is expressed in actively growing tissues, especially in pre-pollination ears at the flowering stage (Pang et al., 2013). In sorghum, *SbABCB16* (Sb06g018860) and *SbABCB18* (Sb06g030350) present the closest protein sequence similarity to *ABCB19* from *Arabidopsis. SbABCB16* expression is highest in the roots and is not responsive to IAA and 1-NOA treatments, while *SbABCB18* is mostly expressed in leaves and is up-regulated by IAA, 1-NOA, and BR applications (Shen et al., 2010).

## AUX/LAXs

The existence of auxin importers in plants was first demonstrated studying the *Arabidopsis auxin insensitive 1* (*aux1*) mutant, which carries defects in roots gravitropic response. *AtAUX1* belongs to a small gene family composed of four highly conserved proteins that share similarities with amino acid transporters: *AtAUX1*, *AtLAX1*, -*2*, and *-3* (Péret et al., 2012). *AtAUX1* encodes a protein similar to fungal amino acid permeases and is expressed in columella, lateral root cap, epidermis, and stele tissues of the primary root where it acts as an auxin importer (Bennett et al., 1996; Swarup et al., 2001, 2004; Carrier et al., 2008). AtAUX1 is involved in auxin-regulated root gravitropic response together with the auxin exporter AtPIN2. The coordinated action of these two proteins forms a lateral auxin gradient which inhibits the expansion of epidermal cells on the lower side of the root relative to the upper side, eventually causing the downward root curvature (Swarup et al., 2005). *aux1*, as well as *pin2 Arabidopsis* mutants are agravitropic and *aux1* also presents a decreased number of lateral roots due to defects in lateral root initiation (Marchant et al., 2002). *AtAUX1* and *AtLAX1,* act redundantly in regulating the phyllotactic pattern in *Arabidopsis* although *AtLAX2* is not expressed in the SAM L1 layer. Since *AtLAX2* is expressed in the forming primordium vasculature, one hypothesis is that AtLAX2 enhances the strength of the primordium as an auxin sink by pulling IAA from the L1 layer of the SAM (Bainbridge et al., 2008; Kierzkowski et al., 2013). *AtLAX2* is involved in vascular development in cotyledons and it is also expressed in young vascular tissues, the quiescent center and columella cells in the primary root (Péret et al., 2012). *AtLAX3* is expressed in the columella and stele of the primary root and it is involved in lateral root development, as *Arabidopsis lax3* mutants show delayed lateral root emergence (Swarup et al., 2008). No root growth–related defects or lateral root–related defects are observed in either *lax1* or *lax2* single mutants while *aux1lax3* double mutant shows a severe reduction in the number of emerged lateral roots (Swarup et al., 2008). Auxin binding and import activity of AUX/LAX proteins has been demonstrated using an oocyte expression system for AtAUX1 (Yang et al., 2006), AtLAX3, and AtLAX1 or a yeast-based heterologous expression system in the case of AtLAX2 (Yang et al., 2006; Carrier et al., 2008; Péret et al., 2012).

### AUX/LAXs IN MONOCOTS

Recently, the expression profile of a putative *AUX1* homolog in rice (*OsAUX1*, Genebank ID AK068536) has been published (Song et al., 2013). The study investigates lateral roots developmental pattern, auxin distribution, PAT and expression of auxin transporter genes in the rice cultivars “Nanguang” and “Elio,” under different nitrogen availability. Expression of *OsAUX1* results higher in the lateral root initiation and emergence zone of “Nanguang” roots in response to partial NO_3_^-^ nutrition rather than to NH_4_^+^ alone. *OsAUX1* is up-regulated in the lateral root elongation zone in the roots of both cultivars in response to phosphorus–nitrogen–nitrogen (PNN) compared to NH_4_^+^ alone (Song et al., 2013).

ZmAUX1, the closest maize homolog of AtAUX1, has 7–10 predicted TMDs and it’s 73% identical to AtAUX1 (Hochholdinger et al., 2000). Northern blot experiments show expression in the tips of primary, lateral, seminal, and crown roots. *In situ* hybridization shows that *ZmAUX1* expression is tissue-specific and confined to the endodermal and pericycle cell layers of the primary root, as well as to the epidermal cell layer (Hochholdinger et al., 2000). ZmAUX1 and AtAUX1 exhibit a preference for IAA and 2,4-D over NAA as substrate and are subject to differential transport inhibition by hexyloxy and benzyloxy derivatives of IAA (Parry et al., 2001; Tsuda et al., 2011). Transcriptome analysis indicates a role for *ZmAUX1* in leaf primordia differentiation, although evidence is still not conclusive (Brooks et al., 2009).

Five *LAX* genes, named *SbLAX1-5*, have been identified in sorghum. The corresponding proteins present a highly conserved core region with 10 predicted transmembrane helices and their transcript levels are higher in leaves and stems rather than in roots and inflorescence tissues (Shen et al., 2010). Expression analysis of 3-weeks-old sorghum seedlings indicates that IAA treatment induces *SbLAX2* and *SbLAX3*, but it inhibits *SbLAX1* and *SbLAX4* expression in leaves and roots, as well as it down-regulates *SbLAX5* expression in leaves. BR treatment induces the expression of all five *SbLAX* genes in roots while it down-regulates *SbLAX1* and *-4* in leaves. ABA, salt, and drought treatments alter the expression profile of all *SbLAXs* (Shen et al., 2010; Wang et al., 2011).

## PILS

**FIGURE 3:**
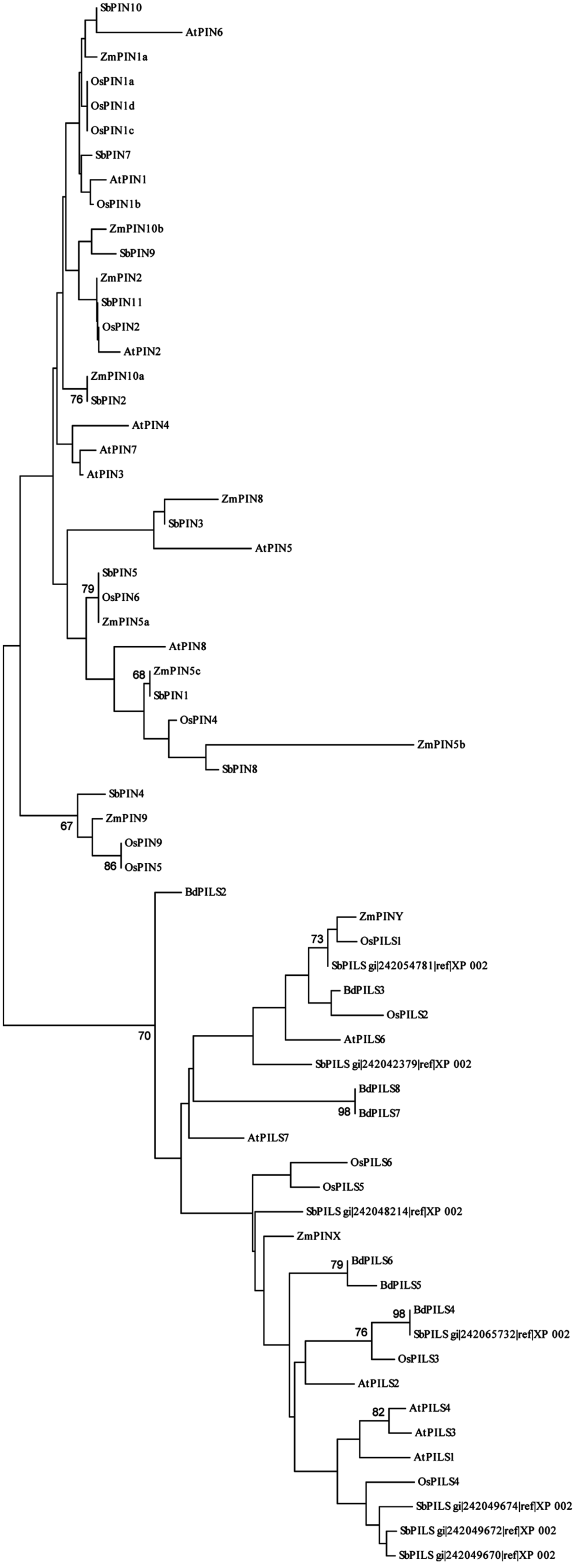
**Neighbor-joining sequence similarity analysis of the PIN and PILS proteins from *Arabidopsis*, rice, maize, sorghum, and *Brachypodium*.** The unrooted tree shows the degree of sequence similarity among PIN and PILS proteins from *Arabidopsis*, rice, maize, sorghum, and *Brachypodium*. ZmPINX and ZmPINY present higher overall similarity to PILS rather than PIN proteins. Bootstrap values higher than 60 are indicated at each node.

PIN-LIKES represent the most recently characterized family of plant auxin transport proteins and include seven members in *Arabidopsis*. PILS show low (10–18%) sequence identity with PINs and they are all capable of transporting auxin across the PM in heterologous systems (Barbez et al., 2012). PILS regulate intracellular auxin accumulation at the ER and thus reduce the availability for free auxin that can reach the nucleus, possibly exerting a role in auxin signaling that is comparable to that of AtPIN5 (Barbez et al., 2012; Barbez and Kleine-Vehn, 2013). The *PILS* family is conserved throughout the plant lineage, having representatives in several taxa including unicellular algae, where *PINs* have not been found yet. This indicates that *PILS* could be evolutionarily older than *PINs* (Feraru et al., 2012; Viaene et al., 2013). Six *PILS* have been identified in rice, 10 in maize, 7 in sorghum, and 8 in *Brachypodium* (**Figure 3**; Feraru et al., 2012). Forestan et al. (2012) identified two maize proteins that in sequence comparison analysis do not cluster with *Arabidopsis* PINs: ZmPINX and ZmPINY. Our sequence comparison verified that the two proteins are more similar to PILS rather than PINs, as previously hypothesized (**Figure 3**). Expression analysis for these genes shows that they are ubiquitously expressed and differentially up-regulated in maize organs. In detail, *ZmPINX* is up-regulated in root apex and male and female inflorescences, while *ZmPINY* is highly expressed during kernel development (Forestan et al., 2012).

## CONCLUSION

Auxin has a fundamental role in plant organs formation and its polar transport across cellular membranes is crucial for the correct development and response to external *stimuli*. Master regulators of PAT are auxin transport proteins, which have been extensively studied in *Arabidopsis* but not in other species, mainly due to the difficulty to obtain loss-of-function mutants. In monocots, only a few of these transporters have been characterized, mainly in rice and maize and most of the information available has been obtained by expression analyses without functional characterization. There are substantial divergences in development and plant structure between monocots and dicots. Differences are present in seed, vascular system, and leaf developmental programs (Tsiantis, 1999; Scarpella and Meijer, 2004; Coudert et al., 2010; Sreenivasulu and Wobus, 2013). The monocot root system architecture and cellular organization also differ considerably from those of dicots (Hochholdinger et al., 2004; Smith and De Smet, 2012). In addition, monocots have a segmented stem as opposed to the unsegmented stem of dicots. Auxin transporter families are larger in monocots allowing for the possibility of functional redundancy, but also for neo- and sub-functionalization of specific proteins. Monocot-specific and organ-specific proteins exist and they have a distinct role in regulating auxin driven organ development (PIN9). In some cases, alterations in PAT result in interesting new traits, such as dwarfism in maize and sorghum *br2* and *dw3* mutants respectively, which can be exploited to generate more productive lines through breeding programs. Moreover, many more short-statured mutants exist in maize that may have defects in auxin transport, although none of these mutants have been characterized in any detail. Interestingly, quite a few of these mutants exhibit dominant inheritance (Johal, unpublished) that makes them interesting in at least two ways. First, they can help side step gene redundancy problems and allow the functional exploration of additional genes. Second, they can be used in MAGIC (mutant-assisted gene identification and characterization)-based enhancer suppressor screens to unveil natural variation in a trait of interest (Johal et al., 2008). Even transgenic reporters for auxin activity can be used *in lieu* of *bona fide* dominant mutants for such MAGIC screens. The traditional enhancer/suppressor screens based on mutagenesis can also be employed to identify additional genes that encode auxin transporters. One such resource already exists in sorghum, where a line carrying a *dw3* mutation in *SbABCB1* was EMS mutagenized to produce and sequence M2 populations for both forward and reverse genetics (Krothapalli et al., 2013). These M2 populations can be screened to identify other genes in the network with the ability to suppress or enhance the *dw3* phenotype. Finally, there is the exciting possibility of using new genome editing and reverse genetics tools such as CRISPR/Cas9, which has been shown to work in rice and maize (Miao et al., 2013; Liang et al., 2014). Technologies like this can be used to alter the expression and function of genes encoding auxin transporters in monocots and this may lead to important new breakthroughs in our understanding of their roles in development and response to the environment.

## Conflict of Interest Statement

The authors declare that the research was conducted in the absence of any commercial or financial relationships that could be construed as a potential conflict of interest.
