# Supplementary material for: The role of auxin transporters in monocots development
Source: Front Plant Sci. 2014 Aug 15;5:393. doi: 10.3389/fpls.2014.00393 (PMC4133927; doi:10.3389/fpls.2014.00393)
Supplement: Supplementary file 1 [file Data_Sheet_1.DOCX]

| **Table 1. List of auxin transporters and putative transporters from Arabidopsis, maize, rice, sorghum and brachypodium. Gene identifier, common name, expression pattern, subcellular localization (if known) and main reference articles are included. E=embryo, S=stem, SB=stem base, R=root, L=leaf, T=shoot, M=male inflorescence, F=female inflorescence, P=panicle, N=node, I=internode, D=seed, O=cob, W=flower, ER=Endoplasmic Reticulum, PM=plasma membrane.** | | | | | | | | | | | |
| --- | --- | --- | --- | --- | --- | --- | --- | --- | --- | --- | --- |
|  |  |  |  |  |  |  |  |  |  |  |  |
|  |  |  |  |  |  |  |  |  |  |  |  |
| **PIN** | | | | | | | | | | | |
|  | | Gene Identifier | | Name | | Expression | | Subcellular localization | References | | |
|  | |  |  |  |  |  |  |  |  |  |  |
| *Arabidopsis thaliana* | | AT1G73590 | | AtPIN1 | | E,S,R | | PM | Friml et al., 2003; Vieten et al., 2005; Zádníková et al., 2010; Bender et al., 2013; Rosquete et al., 2013; Sawchuk et al., 2013; Bennett et al., 2014 | | |
|  |  | AT5G57090 | | AtPIN2 | | E,S,R | | PM |  |  |  |
|  |  | AT1G70940 | | AtPIN3 | | E,S,R | | PM |  |  |  |
|  |  | AT2G01420 | | AtPIN4 | | R(tip) | | PM |  |  |  |
|  |  | AT5G16530 | | AtPIN5 | |  | | PM,ER |  |  |  |
|  |  | AT1G77110 | | AtPIN6 | | R | | ER |  |  |  |
|  |  | AT1G23080 | | AtPIN7 | | E,S,R | | PM |  |  |  |
|  |  | AT5G15100 | | AtPIN8 | | M | | ER |  |  |  |
| *Brachypodium distachyon* | | XM_003563990.1 | | BdPIN1a | |  | |  | O'Connor et al., 2014 | | |
|  |  | XM_003570618.1 | | BpPIN1b | |  | |  |  |  |  |
| *Oryza sativa* | | LOC_Os06g12610 | | OsPIN1a | | R,SB,S,L,P | |  | Wang et al., 2009 | | |
|  |  | LOC_Os02g50960 | | OsPIN1b | | R,SB,S,L,P | |  |  |  |  |
|  |  | LOC_Os11g04190 | | OsPIN1c | | R,SB,S,L,P | |  |  |  |  |
|  |  | LOC_Os12g04000 | | OsPIN1d | |  | |  |  |  |  |
|  |  | LOC_Os06g44970 | | OsPIN2 | | R,SB,P | |  |  |  |  |
|  |  | LOC_Os01g45550 | | OsPIN3a | |  | |  |  |  |  |
|  |  | LOC_Os05g50140 | | OsPIN3b | |  | |  |  |  |  |
|  |  | LOC_Os01g69070 | | OsPIN5a | | R,SB,S,L,P | |  |  |  |  |
|  |  | LOC_Os08g41720 | | OsPIN5b | | R,SB,S,L,P | |  |  |  |  |
|  |  | LOC_Os09g32770 | | OsPIN5c | |  | |  |  |  |  |
|  |  | LOC_Os01g51780 | | OsPIN8 | |  | |  |  |  |  |
|  |  | LOC_Os01g58860 | | OsPIN9 | |  | |  |  |  |  |
|  |  | LOC_Os01g45550 | | OsPIN10a | | SB,S,L,P | |  |  |  |  |
|  |  | LOC_Os05g50140 | | OsPIN10b | | SB,L | |  |  |  |  |
| *Sorghum bicolor* | | Sb02g029210 | | SbPIN1 | | W,S | |  | Shen et al., 2010; Wang et al., 2014 | | |
|  |  | Sb03g029320 | | SbPIN2 | | L,S | |  |  |  |  |
|  |  | Sb03g032850 | | SbPIN3 | | W | |  |  |  |  |
|  |  | Sb03g037350 | | SbPIN4 | |  | |  |  |  |  |
|  |  | Sb03g043960 | | SbPIN5 | | P | |  |  |  |  |
|  |  | Sb04g028170 | | SbPIN6 | | L,W,S,R | |  |  |  |  |
|  |  | Sb05g002150 | | SbPIN7 | |  | |  |  |  |  |
|  |  | Sb07g026370 | | SbPIN8 | |  | |  |  |  |  |
|  |  | Sb10g004430 | | SbPIN9 | | W | |  |  |  |  |
|  |  | Sb10g008290 | | SbPIN10 | | L,W,S,R | |  |  |  |  |
|  |  | Sb10g026300 | | SbPIN11 | | S,R | |  |  |  |  |
| *Zea mays* | | GRMZM2G098643 | | ZmPIN1a | | S,R,F,M,N | |  | Forestan et al.,2012 | | |
|  |  | GRMZM2G074267 | | ZmPIN1b | | S,L,R,F,M,N | |  |  |  |  |
|  |  | GRMZM2G149184 | | ZmPIN1c | | S,R,N | |  |  |  |  |
|  |  | GRMZM2G171702_T01 | | ZmPIN1d | | F,M,N | |  |  |  |  |
|  |  | JQ421085.1 | | ZmPIN2 | | R(apex),F,M | |  |  |  |  |
|  |  | GRMZM2G025742 | | ZmPIN5a | | R(elongation),N | |  |  |  |  |
|  |  | GRMZM2G148648 | | ZmPIN5b | | F,M,N | |  |  |  |  |
|  |  | GRMZM2G040911 | | ZmPIN5c | |  | |  |  |  |  |
|  |  | GRMZM5G839411 | | ZmPIN8 | | F,M,N | |  |  |  |  |
|  |  | GRMZM5G859099 | | ZmPIN9 | | R,N | |  |  |  |  |
|  |  | GRMZM2G126260 | | ZmPIN10a | | F,M,N | |  |  |  |  |
|  |  | GRMZM2G160496 | | ZmPIN10b | | F,M,N | |  |  |  |  |
|  | |  | |  | |  | |  |  | | |
| **AUX/LAX** | | | | | | | | | | | |
|  | | Gene Identifier | | Name | | Expression | | Subcellular localization | References | | |
| *Arabidopsis thaliana* | | AT3G38120 | | AUX1 | | R,S | |  | Swarut et al.,2008; Peret et al.,2010 | | |
|  |  | AT5G01240 | | LAX1 | |  | |  |  |  |  |
|  |  | AT2G21050 | | LAX2 | |  | |  |  |  |  |
|  |  | AT1G77690 | | LAX3 | | R | |  |  |  |  |
| *Oryza sativa* | | LOC_Os01g63770 | | OsLAX1 | |  | |  | Song et al., 2013 | | |
|  |  | LOC_Os03g14080 | | OsLAX2 | |  | |  |  |  |  |
|  |  | LOC_Os05g37470 | | OsLAX3 | |  | |  |  |  |  |
|  |  | LOC_Os10g05690 | | OsLAX4 | |  | |  |  |  |  |
|  |  | LOC_Os11g06820 | | OsLAX5 | |  | |  |  |  |  |
| *Sorghum bicolor* | | Sb01g026240 | | SbLAX1 | | L,S | |  | Shen et al., 2010; Wang et al., 2011 | | |
|  |  | Sb01g041270 | | SbLAX2 | | S | |  |  |  |  |
|  |  | Sb03g040320 | | SbLAX3 | |  | |  |  |  |  |
|  |  | Sb05g004250 | | SbLAX4 | |  | |  |  |  |  |
|  |  | Sb09g021990 | | SbLAX5 | | L,W,S,R | |  |  |  |  |
| *Zea mays* | | GRMZM2G067022 | |  | |  | |  | Hochholdinger et al., 2000; Brooks et al., 2009 | | |
|  |  | GRMZM2G127949 | |  | |  | |  |  |  |  |
|  |  | GRMZM2G045057 | |  | |  | |  |  |  |  |
|  |  | GRMZM2G149481 | |  | |  | |  |  |  |  |
|  |  | GRMZM2G129413 | |  | |  | |  |  |  |  |
|  | |  | |  | |  | |  |  | | |
| **ABCB** | | | | | | | | | | | |
|  |  | Gene Identifier | | Name | | Expression | | Subcellular localization | References | | |
| *Arabidopsis thaliana* | | AT2G36910 | | AtABCB1 | | R,S | | PM | Terasaka et al., 2005; Lewis et al., 2009; Zazimalova et al.,2010; Kubes et al.,2011; Cho et al., 2012; Zhao et al., 2013; Kamimoto et al., 2014 | | |
|  |  | AT4G25960 | | AtABCB2 | |  | |  |  |  |  |
|  |  | AT4G01820 | | AtABCB3 | |  | |  |  |  |  |
|  |  | AT2G47000 | | AtABCB4 | | R,S | | PM |  |  |  |
|  |  | AT4G01830 | | AtABCB5 | |  | |  |  |  |  |
|  |  | AT2G39480 | | AtABCB6 | |  | |  |  |  |  |
|  |  | AT5G46540 | | AtABCB7 | |  | |  |  |  |  |
|  |  | AT3G30875 | | AtABCB8 | |  | |  |  |  |  |
|  |  | AT4G18050 | | AtABCB9 | |  | |  |  |  |  |
|  |  | AT1G10680 | | AtABCB10 | |  | |  |  |  |  |
|  |  | At1g02520 | | AtABCB11 | |  | | PM |  |  |  |
|  |  | AT1G02530 | | AtABCB12 | |  | |  |  |  |  |
|  |  | AT1G27940 | | AtABCB13 | |  | |  |  |  |  |
|  |  | AT1G28010 | | AtABCB14 | | S | |  |  |  |  |
|  |  | AT3G28345 | | AtABCB15 | | S | |  |  |  |  |
|  |  | AT3G28360 | | AtABCB16 | |  | |  |  |  |  |
|  |  | AT3G28380 | | AtABCB17 | |  | |  |  |  |  |
|  |  | AT3G28390 | | AtABCB18 | |  | |  |  |  |  |
|  |  | AT3G28860 | | AtABCB19 | | R,S | | PM |  |  |  |
|  |  | AT3G55320 | | AtABCB20 | |  | |  |  |  |  |
|  |  | AT3G62150 | | AtABCB21 | | R,S | |  |  |  |  |
|  |  | AT3G28415 | | AtABCB22 | |  | |  |  |  |  |
| *Oryza sativa* | | LOC_Os01g18670.1 | | OsABCB1 | |  | |  | Garcia et al., 2004; Knöller et al., 2010; Xu et al., 2014 | | |
|  |  | LOC_Os01g35030.1 | | OsABCB3 | |  | |  |  |  |  |
|  |  | LOC_Os01g50080.1 | | OsABCB4 | |  | |  |  |  |  |
|  |  | LOC_Os01g50100.1 | | OsABCB5 | |  | |  |  |  |  |
|  |  | LOC_Os01g50160.1 | | OsABCB6 | |  | |  |  |  |  |
|  |  | LOC_Os01g52550.1 | | OsABCB7 | |  | |  |  |  |  |
|  |  | LOC_Os01g74470.1 | | OsABCB8 | |  | |  |  |  |  |
|  |  | LOC_Os02g09720.1 | | OsABCB9 | |  | |  |  |  |  |
|  |  | LOC_Os02g46680.1 | | OsABCB11 | |  | |  |  |  |  |
|  |  | LOC_Os03g08380.1 | | OsABCB12 | |  | |  |  |  |  |
|  |  | LOC_Os03g17180.1 | | OsABCB13 | |  | |  |  |  |  |
|  |  | LOC_Os04g40570.1 | | OsABCB15 | |  | |  |  |  |  |
|  |  | LOC_Os05g47490.1 | | OsABCB18 | |  | |  |  |  |  |
|  |  | LOC_Os05g47500.1 | | OsABCB19 | |  | |  |  |  |  |
|  |  | LOC_Os08g05690.1 | | OsABCB20 | |  | |  |  |  |  |
|  |  | LOC_Os08g05710.1 | | OsABCB21 | |  | |  |  |  |  |
|  |  | LOC_Os08g45030.1 | | OsABCB22 | |  | |  |  |  |  |
| *Sorghum bicolor* | | Sb01g039110 | | SbABCB1 | | N | |  | Multani et al., 2003; Schen et al., 2010 | | |
|  |  | Sb02g019540 | | SbABCB2 | |  | |  |  |  |  |
|  |  | Sb03g011860 | | SbABCB3 | |  | |  |  |  |  |
|  |  | Sb03g023740 | | SbABCB4 | |  | |  |  |  |  |
|  |  | Sb03g031990 | | SbABCB5 | |  | |  |  |  |  |
|  |  | Sb03g032000 | | SbABCB6 | | L,R | |  |  |  |  |
|  |  | Sb03g032030 | | SbABCB7 | | R | |  |  |  |  |
|  |  | Sb03g033290 | | SbABCB8 | | L,R | |  |  |  |  |
|  |  | Sb03g047490 | | SbABCB9 | |  | |  |  |  |  |
|  |  | Sb04g006087 | | SbABCB10 | |  | |  |  |  |  |
|  |  | Sb04g006090 | | SbABCB11 | | L,W,R | |  |  |  |  |
|  |  | Sb04g006100 | | SbABCB12 | |  | |  |  |  |  |
|  |  | Sb04g022480 | | SbABCB13 | |  | |  |  |  |  |
|  |  | Sb04g031170 | | SbABCB14 | |  | |  |  |  |  |
|  |  | Sb06g001440 | | SbABCB15 | |  | |  |  |  |  |
|  |  | Sb06g018860 | | SbABCB16 | |  | |  |  |  |  |
|  |  | Sb06g020350 | | SbABCB17 | |  | |  |  |  |  |
|  |  | Sb06g030350 | | SbABCB18 | |  | |  |  |  |  |
|  |  | Sb07g003510 | | SbABCB19 | |  | |  |  |  |  |
|  |  | Sb07g003520 | | SbABCB20 | |  | |  |  |  |  |
|  |  | Sb07g023730 | | SbABCB21 | |  | |  |  |  |  |
|  |  | Sb09g002940 | | SbABCB22 | |  | |  |  |  |  |
|  |  | Sb09g027320 | | SbABCB23 | |  | |  |  |  |  |
|  |  | Sb09g027330 | | SbABCB24 | |  | |  |  |  |  |
| *Zea mays* | | GRMZM2G004748 | | ZmABCB1 | | D | |  |  | | |
|  |  | GRMZM2G025860 | | ZmABCB2 | | L | |  |  | | |
|  |  | G RMZM2G072850 | | ZmABCB3 | | S,I | |  |  | | |
|  |  | GRMZM2G082385 | | ZmABCB4 | | D | |  |  | | |
|  |  | GRMZM2G085111 | | ZmABCB5 | |  | |  |  | | |
|  |  | GRMZM2G085236 | | ZmABCB6 | | T | |  |  | | |
|  |  | GRMZM2G086730 | | ZmABCB7 | | O,D | |  |  | | |
|  |  | GRMZM2G111462 | | ZmABCB8 | | D | |  |  | | |
|  |  | GRMZM2G119894 | | ZmABCB9 | | R | |  |  | | |
|  |  | GRMZM2G125424 | | ZmABCB10 | | O | |  |  | | |
|  |  | GRMZM2G167658 | | ZmABCB11 | | L | |  |  |  |  |
|  |  | GRMZM2G315375 | | ZmABCB12 | | K | |  |  |  |  |
|  |  | GRMZM2G333183 | | ZmABCB13 | | M | |  |  |  |  |
|  |  | GRMZM2G441722 | | ZmABCB14 | | O | |  |  |  |  |
|  |  | GRMZM5G843192 | | ZmABCB15 | | M | |  |  |  |  |
|  |  | GRMZM5G891159 | | ZmABCB16 | | T | |  |  |  |  |
|  |  | GRMZM2G014089 | | ZmABCB17 | | L | |  |  |  |  |
|  |  | AC233882 | | ZmABCB18 | |  | |  | Multani et al., 2003; Knöller et al., 2010; Pang et al., 2013 | | |
|  |  | GRMZM2G158652 | | ZmABCB19 | | D | |  |  |  |  |
|  |  | GRMZM2G006310 | | ZmABCB20 | |  | |  |  |  |  |
|  |  | GRMZM2G049351 | | ZmABCB21 | | T | |  |  |  |  |
|  |  | GRMZM2G072071 | | ZmABCB22 | | L,M | |  |  |  |  |
|  |  | GRMZM2G077361 | | ZmABCB23 | | L | |  |  |  |  |
|  |  | GRMZM2G146034 | | ZmABCB24 | | R | |  |  | | |
|  |  | GRMZM2G153961 | | ZmABCB25 | |  | |  |  | | |
|  |  | GRMZM2G401769 | | ZmABCB26 | | T | |  |  | | |
|  |  | GRMZM2G432390 | | ZmABCB27 | | L | |  |  | | |
|  |  | GRMZM5G833207 | | ZmABCB28 | | R,D | |  |  | | |
|  |  | GRMZM5G843537 | | ZmABCB29 | |  | |  |  | | |
|  |  | GRMZM2G388539 | | ZmABCB30 | | L,D | |  |  | | |
|  |  | GRMZM2G032936 | | ZmABCB31 | | L,M | |  |  | | |
|  |  |  |  |  |  |  |  |  |  |  |  |
| **PILS** | | | | | | | | | | | |
|  | | Gene Identifier | | Name | | Expression | | Subcellular localization | References | | |
|  | |  |  |  |  |  |  |  |  |  |  |
| *Arabidopsis thaliana* | |  | AT1G20925 | AtPILS1 | |  |  |  | Forestan et al., 2012; Feraru et al., 2014 | | |
|  |  |  | AT1G71090 | AtPILS2 | |  |  |  |  |  |  |
|  |  |  | AT1G76520 | AtPILS3 | |  |  |  |  |  |  |
|  |  |  | AT1G76530 | AtPILS4 | |  |  |  |  |  |  |
|  |  |  | AT2G17500 | AtPILS5 | |  |  |  |  |  |  |
|  |  |  | AT5G01990 | AtPILS6 | |  |  |  |  |  |  |
|  |  |  | AT5G65980 | AtPILS7 | |  |  |  |  |  |  |
| *Brachypodium distachyon* | |  | Bdi1G63446 | BdPILS1 | |  |  |  | Feraru et al., 2014 | | |
|  |  | gi\|357120862\|ref\|XM_003562096.1\| | | BdPILS2 | |  |  |  |  |  |  |
|  |  | gi\|357125608\|ref\|XM_003564436.1\| | | BdPILS3 | |  |  |  |  |  |  |
|  |  | gi\|357126973\|ref\|XM_003565114.1\| | | BdPILS4 | |  |  |  |  |  |  |
|  |  | gi\|357138294\|ref\|XM_003570683.1\| | | BdPILS5 | |  |  |  |  |  |  |
|  |  | gi\|357144102\|ref\|XM_003573124.1\| | | BdPILS6 | |  |  |  |  |  |  |
|  |  | gi\|357159092\|ref\|XM_003578288.1\| | | BdPILS7 | |  |  |  |  |  |  |
|  |  | gi\|357159092\|ref\|XM_003578564.1\| | | BdPILS8 | |  |  |  |  |  |  |
| *Oryza sativa* | | gi\|115440694\|ref\|NM_001051162.1\| | | OsPILS1 | |  |  |  | Feraru et al., 2014 | | |
|  |  | gi\|115464530\|ref\|NM_001062400.1\| | | OsPILS2 | |  |  |  |  |  |  |
|  |  | gi\|115475144\|ref\|NM_001067704.1\| | | OsPILS3 | |  |  |  |  |  |  |
|  |  | gi\|297727104\|ref\|NM_001188987.1\| | | OsPILS4 | |  |  |  |  |  |  |
|  |  | gi\|115480586\|ref\|NM_001070422.1\| | | OsPILS5 | |  |  |  |  |  |  |
|  |  | gi\|297609931\|ref\|NM_001070423.2\| | | OsPILS6 | |  |  |  |  |  |  |
| Zea mays | |  | ZmGSStuc11-12-04.463.1 | ZmPINX | | S,L,R(apex),M,F | |  | Forestan et al., 2012 | | |
|  |  |  | GRMZM2G050089 | ZmPINY | | S,L,R(apex),M,F | |  |  |  |  |
